# *In vivo* functional screening on innate immunity of lactic acid bacteria in *Galleria mellonella* preclinical model: comparative analysis of *Lactiplantibacillus plantarum* and *Lentilactobacillus kefiri*

**DOI:** 10.3389/fcimb.2025.1681687

**Published:** 2025-10-10

**Authors:** Antonio Guarnieri, Marilina Falcone, Natasha Brancazio, Farwa Mukhtar, Addis Temie Worku, Marco Alfio Cutuli, Vincenzo Pio Iacovino, Sonia Ganassi, Antonio De Cristofaro, Roberto Di Marco, Giulio Petronio Petronio

**Affiliations:** ^1^ Department of Medicina e Scienze della Salute “V. Tiberio”, Università degli Studi del Molise, Campobasso, Molise, Italy; ^2^ Department of Agricultural, Environmental and Food Sciences, Università degli Studi del Molise, Campobasso, Molise, Italy; ^3^ Department of Drug and Health Sciences, Università degli Studi di Catania, Catania, Sicily, Italy

**Keywords:** *Lactiplantibacillus plantarum*, *Lentilactobacillus kefiri*, *Galleria mellonella*, larva, innate immunity recognition systems, immunomodulation, probiotic-mediated host defense

## Abstract

**Introduction:**

Among probiotics, Lactic Acid Bacteria modulate host immunity via strain-specific molecular patterns. The invertebrate model *Galleria mellonella* offers conserved innate immune pathways and is increasingly applied for preclinical screening of probiotic functions.

**Methods:**

We evaluated the immunomodulatory activity of *Lactiplantibacillus plantarum* ATCC 14917 and *Lentilactobacillus kefiri* DSM 10551 in *G. mellonella*. Larvae were injected with 10^6^ CFU/larva of each strain, and survival and health indices were monitored for 72 h. The temporal transcriptional response of ten innate immunity-related genes, including Toll and IMD signalling, Toll receptor, cytokine-like ligand, and antimicrobial effectors, was assessed via qRT-PCR over 3–24 hours, complemented by correlation and hierarchical clustering to identify co-expression modules and strain-specific transcriptional patterns.

**Results:**

Both strains were non-toxic and induced strain-dependent gene expression patterns. *L. plantarum* induced a stronger and more sustained activation of immune signalling pathways and effector responses, whereas *L. kefiri* was characterised by an earlier and prolonged activation of stress-related and tissue-protective mechanisms. Correlation and clustering analyses revealed distinct co-expression modules that reflect modulation of the Toll and IMD pathways.

**Discussion:**

These findings suggest that *G. mellonella* could serve as a cost-effective *in vivo* model for functional screening of *Lactobacillus* spp. with immunomodulatory potential and possible translational relevance to human innate immunity.

## 1 Introduction

The immunomodulatory properties of probiotics have been extensively studied. These microorganisms modulate both innate and adaptive immune responses. In the adaptive branch, they are known to promote regulatory T cell expansion and influence the Th1/Th2 cytokine profile. Their effects on innate immunity are mediated through interactions with mucosal epithelial cells and the activation of pattern recognition receptors expressed by innate immune cells, resulting in downstream signalling and cytokine modulation (Ashaolu et al., 2025).

Among probiotics, Lactic Acid Bacteria (LAB) exert their innate immunomodulatory effects through strain-specific mechanisms involving structural components such as peptidoglycans, lipoteichoic acids (LTAs), teichoic acids, and exopolysaccharides (EPSs) (Rahbar Saadat et al., 2019). These molecules are recognised by pattern recognition receptors (PRRs), including Toll-like receptors (TLRs), NOD-like receptors (NLRs), and C-type lectins, on mucosal epithelial cells, macrophages, and dendritic cells. The engagement of these receptors activates intracellular signalling pathways, particularly NF-κB and MAPK, thereby finely tuning cytokine production (H. G. Kim et al., 2008). Depending on the strain and host context, LAB can suppress pro-inflammatory cytokines, such as TNF-α, IL-6, and IL-8, while enhancing anti-inflammatory mediators, like IL-10 and TGF-β. Additionally, many strains stimulate the secretion of antimicrobial peptides (AMPs), including human β-defensins and cathelicidins (Martín et al., 2014). Through these actions, LABs help maintain immune homeostasis, mitigate excessive inflammation, and reinforce first-line defences against pathogens (Cesare et al., 2020; Adejumo et al., 2023).

LAB, particularly those previously grouped under the broad genus *Lactobacillus*, are widely studied for their health-promoting and immunomodulatory properties. Following a major taxonomic revision based on whole-genome phylogenetics, the genus *Lactobacillus* was reclassified into 25 new genera to reflect better genetic and functional diversity (Zheng et al., 2020). Among these, the two genera *Lactiplantibacillus* and *Lentilactobacillus* represent two distinct lineages with well-characterised probiotic potential. *Lactiplantibacillus plantarum* is well-known for its ability to colonise diverse human-associated niches, particularly the gastrointestinal tract. Within the gut, it contributes to maintaining epithelial barrier integrity, modulates immune responses, and has shown therapeutic potential in various inflammatory and immune-mediated conditions, including inflammatory bowel disease, atopic dermatitis, and oral dysbiosis (Chatsirisakul et al., 2025). *Lentilactobacillus kefiri* typically isolated from fermented dairy products such as kefir, has recently gained attention for its strain-specific immunomodulatory and antimicrobial properties at mucosal surfaces. Studies have shown that *L. kefiri* exerts anti-inflammatory effects in both *in vitro* and *in vivo* models, including LPS-stimulated intestinal epithelial cells and murine models of colitis (Manna et al., 2023).

The increasing clinical and commercial interest in probiotics has underscored the need for rigorous functional validation and clear regulatory frameworks. In the EU, probiotics marketed as foods or supplements are regulated by Regulation (EC) No 1924/2006 (Regulation - 1924/2006 - EN - EUR-Lex, n.d), which does not require clinical trials for commercialisation. However, any health claim must be supported by robust scientific evidence, typically including randomised controlled trials (RCTs) assessed by EFSA (EFSA Panel on Dietetic Products, Nutrition and Allergies (NDA), 2016) (EFSA Panel on Dietetic Products, Nutrition and Allergies (NDA), 2021). Increasingly, probiotics are being developed as therapeutic agents, subject to regulatory requirements similar to those applied to biologic drugs, including well-defined manufacturing processes, strict quality control, and comprehensive preclinical and clinical evidence of efficacy and safety, leading to the classification of these products as Live Biotherapeutic Products (LBPs) (Garg et al., 2024; Spacova et al., 2023).

To meet these expectations, there is a growing emphasis on generating mechanistic data that elucidate how specific strains modulate host immune responses. This includes, but is not limited to, the identification of molecular effectors, their interaction with pattern recognition receptors on immune cells, and the downstream modulation of immune pathways. On the other hand, international regulatory frameworks aimed at reducing the use of vertebrate animals in research, such as the European Directive 2010/63/EU and OECD guidelines (Directive - 2010/63 - EN - EUR-Lex, n.d). These frameworks increasingly emphasise the need for alternative, ethically sustainable models in preclinical testing (Cutuli et al., 2021; Petronio Petronio et al., 2022; Venditti et al., 2021).

In recent years, *Galleria mellonella*, the greater wax moth, has emerged as a valuable *in vivo* model for investigating host–microbe interactions and validating immunomodulatory effects (Cutuli et al., 2019). While mammalian innate immunity has been thoroughly studied, insect immunity—particularly outside the extensively used *Drosophila melanogaster*—remains less explored. Notably, probiotic administration in *Drosophila* has been shown to enhance survival (Erkosar et al., 2013); similarly, probiotics such as *E. coli Nissle* 1917 (EcN1917), have also been reported to improve survival and modulate immune responses in *G. mellonella* challenged with pathogens (Menta et al., 2024). *G. mellonella* presents unique advantages, including thermotolerance at 37 °C and a more complex innate immune architecture. Despite lacking adaptive immunity, *G. mellonella* exhibits a highly organised and pathogen-responsive innate system comprising both cellular and humoral branches (Cutuli et al., 2019). Hemocytes mediate phagocytosis, encapsulation, coagulation, and signalling, and are rapidly recruited upon infection to generate reactive oxygen and nitrogen species (Bergin et al., 2005). In parallel, humoral defences are activated through the phenoloxidase cascade, leading to pathogen melanisation (Bergin et al., 2005), and the transcriptional induction of antimicrobial peptides such as gallerimycin and gloverin via conserved Toll and IMD pathways (Vogel et al., 2011). These integrated immune responses position *G. mellonella* as a robust and translationally relevant model for studying host-pathogen dynamics.

This study aims to employ *G. mellonella* as an alternative *in vivo* model to characterise the immunomodulatory effects of *L. plantarum* ATCC 14917 (LP) and *L. kefiri* DSM 10551 (LK) by analysing the time-course expression of a selected panel of innate immunity-related genes. The analysis focused on central regulators of the Toll and IMD signalling cascades, such as the embryonic polarity protein dorsal (*Dorsal*), relish (*Rel*), and caudal type homeobox (*cad*), as well as Toll receptor 7 (*18w*), and the cytokine-like ligand Spaetzle domain-containing protein (*spz4*). The study also considered antimicrobial effectors, including gallerimycin (*gallerimycin*) and gloverin (*gloverin*), the oxidative enzyme NADPH oxidase 4-like (*NADPH oxidase 4-like*) involved in ROS-mediated microbial killing, and immune effector genes associated with tissue protection, zonadhesin (*IMPI*) and phagocytosis Nck-associated protein 1 Hem (*Hem)*. This gene panel provides a comprehensive overview of the host’s immune activation profile in response to LAB administration, encompassing humoral, oxidative, and cellular responses. These findings aim to define the strain-specific immunomodulatory effects of LP and LK, and to further validate *G. mellonella* as an effective alternative model for the functional screening of LAB with potential clinical applications.

## 2 Methods

### 2.1 Chemicals and reagents

De Man, Rogosa, and Sharpe (MRS) medium was purchased from Liofilchem and prepared according to the manufacturer’s instructions (62g/L, pH 6.2).

TRIzol Reagent User Guide (Invitrogen, Waltham, MA, USA).

cDNA reverse transcription kit (Applied Biosystems™) with RNAse inhibitor.

qRT-PCR was performed using PowerUp™ SYBR™ Green Master Mix (Applied Biosystems). Water, sterile, and molecular biology grade (DEPC-treated, nuclease-, and protease-free) was purchased from HiMedia.

### 2.2 Bacterial strains cultivation and *inocula* preparation

*L. kefiri* DSM 10551 (LK), *L. plantarum* ATCC 14917 (LP), were stored at -80 °C with 20% glycerol. After thawing, the LAB strains were streaked onto MRS agar plates and incubated at 37 °C under microaerophilic conditions for 24–72 hours, until visible growth was observed. Subsequently, a single colony from each plate was transferred into MRS broth and cultured under the same conditions. Bacterial cultures were pelleted and suspended in saline solution to an optical density of 0.15 at 600 nm (PerkinElmer Wallac Victor 1420 Multilabel), corresponding to 1.3 ± 0.2 × 10^^8^ CFU/ml (as determined by the plate count method).

### 2.3 *G. mellonella* larvae acquisition and rearing

*G. mellonella* larvae were purchased from a local supplier (Arcobaleno Pesca Sport, Campobasso, Italy) and used to establish a colony in the laboratory of Agricultural Entomology (Department of Agricultural, Environmental and Food Sciences, University of Molise, Italy). Rearing was conducted in glass containers (40cm height × 20cm diameter) at 28 °C and 40% relative humidity, under complete darkness (0L:24D). The larvae were reared on an artificial diet obtained by combining several protocols (Firacative et al., 2020; Jorjão et al., 2018; Metwally et al., 2012; Mohamed and Amro, 2022; Xue et al., 2023), and was composed of the following ingredients: 300g soy flour (30%), 150g cornmeal (15%), 150g type 1 wheat flour (15%), 100g milk powder (10%), 48g yeast extract (4.8%), 150g glycerol (15%), 100g inverted sugar syrup (10%), 2g multivitamin supplement (0.2%). For all experiments, last-instar larvae, each weighing 270–330 mg, without any grey markings, were used, as described by Fuchs et al. (2010) (Fuchs et al., 2010).

### 2.4 *Lactobacillus* injection in *G. mellonella*

Serial dilutions (10^4^ to 10^6^ CFU/larvae) of each bacterial *inoculum* were administered by injection according to Vertyporokh and Wojda, 2020, with some modifications (Vertyporokh and Wojda, 2020). Briefly, larvae were starved for 48h before injection. Larval abdominal surfaces were disinfected with 70% ethanol and then placed at 4 °C for 5 min to induce cold anaesthesia. A 10µl volume was injected into the last left pseudoleg using a 0.5ml insulin syringe (BD Micro-Fine; needle size: 0.33mm × 12.7mm). Larvae were randomly assigned to experimental groups and injected with either serial dilutions of each bacterial inoculum or with PBS alone (Control group). Following injection, the larvae were incubated at 37 °C in the dark under starvation conditions and monitored at regular intervals for 72 hours. All manipulations were performed under sterile conditions. To confirm the efficacy of the disinfection procedure, swabs from larval cuticles were plated on CLED agar and incubated at 37 °C for 48h.

### 2.5 Survival monitoring and health index assessment

In order to evaluate the sub-lethal inoculum concentration of the *Lactobacillus* strains, larval survival was monitored every 24 hours up to 72 hours. Larvae were considered dead when they showed no signs of movement upon gentle stimulation with forceps. The health status of *G. mellonella* larvae was assessed using a health index score, adapted from Loh et al., 2013 (Loh et al., 2013) and modified to reflect responses to probiotic bacterial treatment. The index evaluates four observable parameters: motility, cocoon formation, degree of melanisation, and survival. Each parameter was scored based on defined behavioural and morphological criteria (**Table 1**). The maximum score for a healthy larva was 10. Observations were carried out at 24, 48, and 72 hours post-injection. Scoring was performed independently by two blinded observers to ensure objective evaluation. Based on the health index score results, a concentration of 10^5^ CFU/larvae was adopted for all subsequent assays.

**Table 1:** *G. mellonella* health index score (Loh et al., 2013).

Category	Description	Score
Activity	No activity	*0*
	Minimal activity upon stimulation	*1*
	Active when stimulated	*2*
	Active without stimulation	*3*
Cocooning	No cocoon	*0*
	Partial cocoon	*0.5*
	Full cocoon	*1*
Melanisation	Complete melanisation	*0*
	≥40% body surface melanised	*1*
	20–40% melanised	*2*
	<20% melanised	*3*
	No melanisation	*4*
Survival	Dead	*0*
	Alive	*2*

### 2.6 *In silico* alignment of *G. mellonella* immune-related proteins with putative human orthologs

To investigate potential orthology between *G. mellonella* immune-related proteins and their human counterparts, a sequential analytical workflow was applied. Primer sequences employed for gene expression analysis (**Table 2**) were used to retrieve *G. mellonella* mRNA via NCBI Primer-BLAST Database Refseq mRNA (*G. mellonella* NCBI: txid 7137). The resulting nucleotide sequences were translated into protein sequences using the NCBI RefSeq database. Each *G. mellonella* protein sequence was aligned against the *Homo sapiens* proteome (NCBI: txid 9606) using BLASTP with default parameters. Where a significant alignment was identified, the aligned region was extracted for downstream analysis and the BLOSUM62 substitution matrix, which represents the standard for protein homology searches (Henikoff and Henikoff, 1992; Mount, 2004). When no significant homology was detected, the full-length *G. mellonella* protein was retained. Pairwise alignments between query and subject proteins were subsequently parsed column by column to derive a conservative consensus sequence. Identical residues were directly included in the consensus, while conservative substitutions—classified according to major physicochemical groups (hydrophobic, positively charged, negatively charged, polar uncharged, and special)—were resolved by retaining the residue present in the query sequence. Non-conservative substitutions were annotated as “X” to indicate ambiguity, and gaps were represented by a retained residue when present or by “–” in the case of double gaps. This rule-based parsing ensured that consensus sequences captured conserved and functionally relevant features across all aligned regions (Capra and Singh, 2007). Consensus sequences were analyzed for conserved domain annotation and architectures, protein family assignments, and inferred molecular functions using the InterPro database (release 106.0, June 19, 2025). All alignment files (FASTA format) are available in the Supplementary Materials, whereas consensus sequences and InterPro analysis files (JSON format) are available in the data repository (10.6084/m9.figshare.29655497).

**Table 2:** Primer sequences used for the target genes in this study.

Gene symbol	Gene description	Accession number (mRNA)	LOC ID	Primer sequence (5’ to 3’)	Reference
*eEF1alpha1*	*elongation factor 1-alpha*	*XM_026903443.1*	*LOC113518486*	*Fw: AACCTCCTTACAGTGAATCC* *Rv: ATGTTATCTCCGTGCCAG*	(Melo et al., 2013)
*Cad*	*cad type homeobox*	*XM_026909593.3*	*LOC113523598*	*Fw: GCAAGAGCGGGCAATACGG* *Rv: CTCGCCTGGTTCTCTTTTCGG*	(Sarvari et al., 2020)
*Dorsal*	*embryonic polarity protein Dorsal*	*XM_052899051.1*	*LOC113512577*	*Fw: CGACGAATTTCTACCTGTAAACC* *Rv: CACCAAACCTTTACTCGACATTT*	(Sarvari et al., 2020)
*spz4*	*Spaetzle domain-containing protein 4*	*XM_031908808.2*	*LOC113517666*	*Fw: AAAGAAGAAATGCAAGCTCCA* *Rv: AACTTAAACCAGTCAGCGAAG*	(Sarvari et al., 2020)
*Rel*	*Rel*	*XM_052893440.1*	*LOC113515637*	*Fw: TCCAAAAAGCACCCTACAATCG* *Rv: GCACTTCGTAGCTCACATCTC*	(Sarvari et al., 2020)
18w*	*Toll-like receptor 7*	*XM_026893057.3*	*LOC113509667*	*Fw: CACTCGATTTAGGCAACAACA* *Rv: TCCGAGACGATCAACACTTC*	(Melo et al., 2013)
gallerimycin *	*gallerimycin -like*	*XM_026909420.3*	*LOC113523440*	*Fw: AACCATCACCGTCAAGCCA* *Rv: TCGAAGACATTGACATCCATTGA*	(Sarvari et al., 2020)
gloverin *	*gloverin*	*XM_026909162.3*	*LOC113523269*	*Fw: AGATGCACGGTCCTACAG* *Rv: GATCGTAGGTGCCTTGTG*	(Melo et al., 2013)
NADPH oxidase 4-like*	*NADPH oxidase 4-like*	*XM_026901959.3*	*LOC113517309*	*Fw: GCTTGACATTGAGCTGTCCA* *Rv: CCGTCCAATCACCTTTGACT*	(Melo et al., 2013)
*Hem*	*Nck associated protein 1 Hem*	*XM_026899938.2*	*LOC113515675*	*Fw: ATTGCTAGCCAGGTTCAGGA* *Rv: AGCTATTTGGCGGAAACTCA*	(Melo et al., 2013)
IMPI *	*zonadhesin*	*XM_052897463.1*	*LOC113511802*	*Fw: TAGTAAGCAGTAGCATAGTCC* *Rv: GCCATCTTCACAGTAGCA*	(Melo et al., 2013)

Genes marked with an asterisk (*) do not have an official gene symbol and are indicated with a generic name; the official identifier is given by the corresponding LOC number.

### 2.7 RNA extraction, cDNA amplification, and qRT-PCR gene expression

3, 6, 12, 18, and 24 hours after *Lactobacillus* infections, *G. mellonella* larvae were anesthetised at 4°C for 10 min. Subsequently, the last part of the larva’s abdomen was cut off using a sterile surgical blade (Swann Morton Limited, Sheffield S6 2BJ, England). Hemolymph was collected into 1.5ml Eppendorf tubes, and the samples were maintained on ice to prevent melanisation (Moya-Andérico et al., 2020). RNA was extracted from the hemolymph of each larval group according to the TRIzol Reagent protocol (Invitrogen, Waltham, MA, USA). A high-capacity cDNA reverse transcription kit (Applied Biosystems) with RNase inhibitor was used for cDNA amplification, following the manufacturer’s instructions. Quantitative real-time PCR (qRT-PCR) was performed using 50µl of PowerUp™ SYBR™ Green Master Mix (Applied Biosystems) with an annealing temperature of 60°C for 15s for 35 cycles, followed by the melt curve analysis (68–95°C). Primer sequences are shown in **Table 2**. Results were normalised against the housekeeping gene *eEF1alpha1* and shown as relative values compared with PBS-treated larvae (Control group) using the ΔΔCt method. For each treatment and time point, gene expression was quantified from three biological replicates, each analysed in duplicate. The complete raw dataset (Ct values) is publicly available at 10.6084/m9.figshare.29655497.

### 2.8 Statistical analysis

The data collected in this study were analysed using statistical software (R version 4.4.0). The adequacy of normal data distribution was assessed using the Shapiro-Wilk test, with a significance level set at *p* < 0.05. For normally distributed values, one-way analysis of variance (ANOVA) was applied with a significance level set at *p* < 0.05 to assess differences between groups, followed by Tukey’s *post hoc* test to identify pairwise significance. Levene’s test was used to confirm the assumption of homogeneity of variances. For non-normally distributed data, the non-parametric Kruskal-Wallis test was applied with a significance level set at *p* < 0.05, followed by Dunn’s *post hoc* test with Bonferroni correction for multiple comparisons. Spearman’s rank correlation coefficient was employed to assess the correlation between gene expression levels. Comprehensive statistical results are accessible in the data repository (10.6084/m9.figshare.29655497).

## 3 Results

### 3.1 *G. mellonella* survival and health status

No mortality was observed in any experimental group (LP, LK, or control) during the 72-hour observation period. Furthermore, larvae did not exhibit signs of distress, such as melanisation, reduced mobility, or impaired cocoon formation (**Table 1**). The health index scores remained consistently high across all groups, with values close to the maximum of 10, our findings align with those of Rossoni et al. (2017) (Rossoni et al., 2017). On this basis, we selected an inoculum of 1.3 ± 0.2×10^5^ CFU/larvae for subsequent assays.

### 3.2 *In silico* alignment and conserved domain functional annotation of *G. mellonella* immune-related proteins

Selected immune-related proteins from *G. mellonella* were aligned with *Homo sapiens* protein sequences (**Supplementary Table S1**). Key alignment metrics were recorded (Bit score, query coverage, E-value, and identity percentage), with the Bit Score used as the primary indicator of alignment strength according to Harbor et al., 2004 (Mount, 2004).

The strongest alignment was observed between membrane-associated protein Hem (XP_026755739.2) and human NCKAP1 (NP_038464.1), with a Bit score of 1459, 99% query coverage, an E-value of 0.0, and 61.85% identity. NADPH oxidase 4-like isoform X1 (XP_026757760.2) aligned with human NOX4 isoform a (NP_058627.2), yielding a Bit score of 281, 73% query coverage, an E-value of 1e-85, and 33.95% identity.

Among transcription factors, embryonic polarity protein Dorsal (XP_052755011.1) aligned with human NF-κB p65 (NP_068810.3), producing a Bit score of 242, 37% query coverage, an E-value of 7e-70, and 45.14% identity. The alignment between NF-κB p110 isoform X1 (XP_052749400.1) and human NF-κB p100 isoform a (NP_001070962.1) resulted in a Bit score of 179, 31% query coverage, an E-value of 4e-45, and 37.96% identity.

The homeobox protein CDX-2 isoform X2 (XP_026765394.1) and human CDX-2 isoform 1 (NP_001256.4) comparison produced a Bit score of 137, 36% query coverage, an E-value of 6e-38, and 79.07% identity. The 18-wheeler/TLR7-like protein (XP_026748858.2) aligned with human SLIT1 (AAI46762.1), yielding a Bit score of 177, 61% query coverage, an E-value of 2e-43, and 26.51% identity.

At the lowest level of similarity, the inducible metalloproteinase inhibitor protein (XP_052753423.1) and human mucin-6 isoform X1 (XP_054187951.1) produced a Bit score of 43.1, 22% query coverage, an E-value of 0.015, and 22.22% identity.

Notably, protein spaetzle (XP_031764668.1), gloverin (XP_026764963.2), and gallerimycin (XP_026765221.2) did not yield any significant alignments with human sequences; no relevant BLAST hits were retrieved for these proteins. Nevertheless, insect-specific domains and protein families were identified through InterPro analysis, indicating their involvement in immune functions, as reported in **Supplementary Table S1**.

### 3.3 Differential modulation of immune-related genes by *L. plantarum* ATCC 14917 and *L. kefiri* DSM 10551

#### 3.3.1 Time course qRT-PCR

Time course qRT-PCR was used to assess the temporal expression profiles of selected immune-related genes in *G. mellonella* larvae following administration of LP or LK.

**Figure 1:**
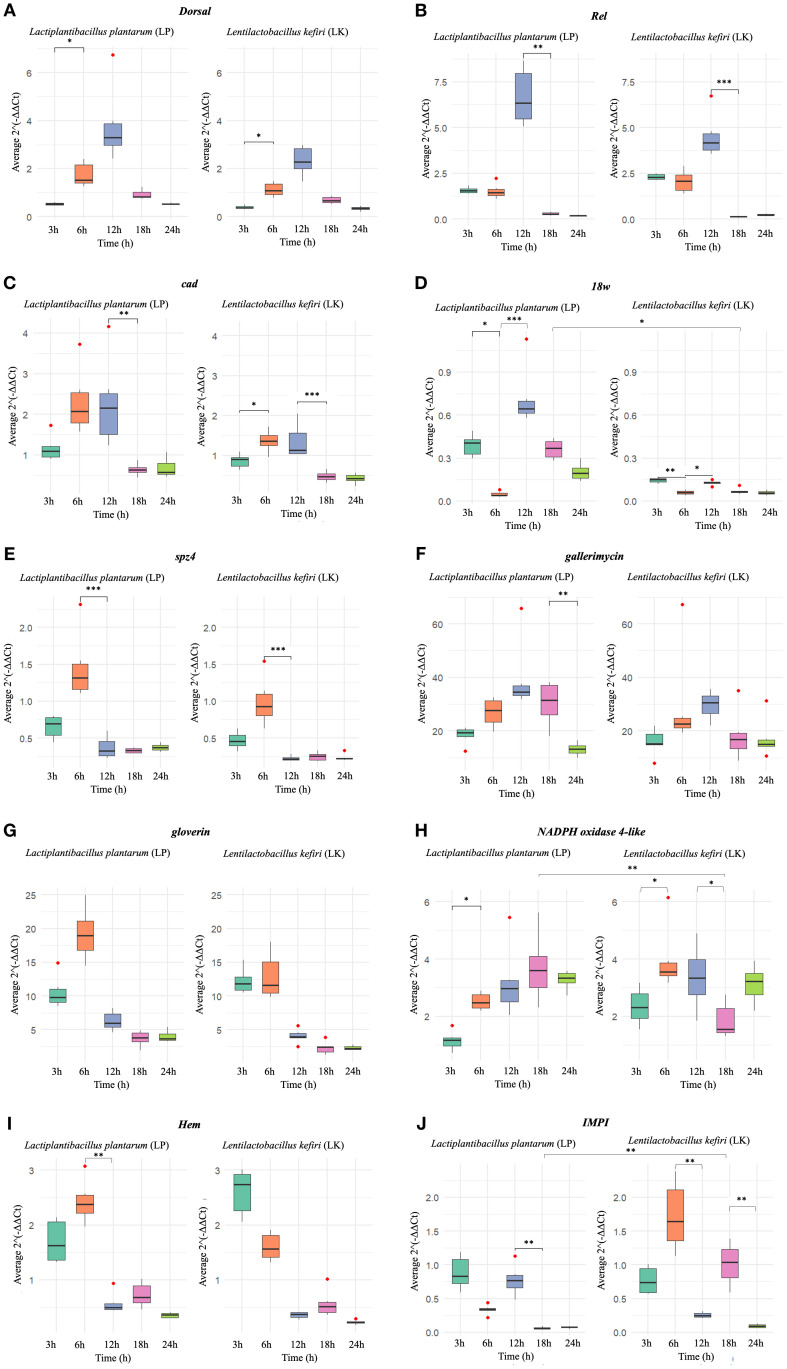
Relative expression (2^-ΔΔCt) of the analysed genes in *G. mellonella* **(A)** dorsal **(B)** *Rel* **(C)** *cad* **(D)** *18w Rel* **(E)** *spz4* **(F)** *gallerimycin* **(G)** *gloverin* **(H)** NADPH oxidase 4 **(I)** *Hem* **(J)** *IMPI*, following administration of *L. plantarum* (LP, right panels) and *L. kefiri* (LK, left panels), at five time points (3h, 6h, 12h, 18h, 24h). Statistically significant differences are shown only between consecutive time points within the same treatment, and between different treatments (LP vs LK) at the same time point (*p < 0.05 **p < 0.01 ***p < 0.001). Outlier data are indicated by red dots.

The results revealed distinct patterns of gene modulation between the two probiotic strains across the different time points (3, 6, 12, 18, and 24 hours post-administration). The fold changes (FC) in gene expression and their statistical significance are summarised in **Figure 1**.

The *Dorsal* gene exhibited an early and transient expression pattern in both treatments, peaking at 12h and declining thereafter (**Figure 1A**). In LP-treated larvae, expression significantly increased from 3h (M = 0.532; SD = 0.059; median = 0.530), indicating mild downregulation (0.5 ≤ FC < 1), to 6h (M = 1.738; SD = 0.502; median = 1.530), indicating mild upregulation (1 < FC ≤ 2) (*p* < 0.05), and reached a maximum at 12h (M = 3.775), corresponding to strong upregulation (FC > 2), followed by a marked decline at 18h and 24h. Similarly, in LK-treated larvae, expression rose from 3h (M = 0.390; SD = 0.068; median = 0.375), reflecting strong downregulation (FC < 0.5), to 6h (M = 1.127; SD = 0.290; median = 1.085), corresponding to mild upregulation (1 < FC ≤ 2) (*p* < 0.05), with a peak at 12h (M = 2.320; strong upregulation) and a subsequent decline. Although the temporal profiles were comparable, LP-treated larvae consistently displayed higher absolute expression levels, with no statistically significant differences between treatments at corresponding time points.

The *Rel* gene displayed its highest expression at 12h in both treatments (**Figure 1B**). In LP-treated samples, expression reached a maximum of M = 6.677 (SD = 1.526; median = 6.340), corresponding to strong upregulation (FC > 2), followed by a significant decrease at 18h (M = 0.297; SD = 0.085; median = 0.275), indicating strong downregulation (FC < 0.5) (*p* < 0.01). A similar pattern was observed in LK-treated larvae, with a peak at 12h (M = 4.512; SD = 1.171; median = 4.155; strong upregulation) and a significant drop at 18h (M = 0.113; SD = 0.033; median = 0.125; strong downregulation) (*p* < 0.001). Although the temporal expression profiles were comparable, LP treatment consistently induced higher expression levels at all time points, with no statistically significant differences between LP and LK at corresponding time points.

The *cad* gene exhibited a comparable temporal expression trend in both treatments, peaking at 6h (**Figure 1C**). In LP-treated larvae, expression reached its maximum at 6h (M = 2.305; SD = 0.799; median = 2.070), corresponding to strong upregulation (FC > 2), followed by a significant decline between 12h and 18h (p < 0.01), reaching mild downregulation at 18h (M = 0.638; SD = 0.145; median = 0.625; 0.5 ≤ FC < 1). In contrast, LK-treated larvae showed a significant increase from 3h (M = 0.867; SD = 0.176; median = 0.905; mild downregulation) to 6h (M = 1.362; SD = 0.262; median = 1.360; mild upregulation) (p < 0.05), with levels remaining elevated until 12h, and then sharply decreasing to strong downregulation at 18h (M = 0.475; SD = 0.125; median = 0.465; FC < 0.5) (*p* < 0.001). Although no significant differences were detected between LP and LK at individual time points, absolute expression values were consistently higher in the LP group.

A biphasic expression pattern of the *18w* gene was observed in both treatments, with an early induction at 3h, a decline at 6h, and a secondary increase at 12h (**Figure 1D**). In LP-treated larvae, the first peak at 3h (M = 0.390; SD = 0.075; median = 0.405) corresponded to strong downregulation (FC < 0.5), followed by a significant decrease at 6h (M = 0.048; SD = 0.018; median = 0.040; strong downregulation) (*p* < 0.05). At 12h, a new statistically significant increase was recorded (M = 0.720; SD = 0.206; median = 0.645; mild downregulation) (*p* < 0.001), after which expression progressively declined. A similar biphasic trend was observed in LK-treated larvae, with a first peak at 3h (M = 0.145; SD = 0.016; median = 0.150; strong downregulation), followed by a significant reduction at 6h (M = 0.060; SD = 0.015; median = 0.060; strong downregulation) (*p* < 0.01), and a secondary increase at 12h (M = 0.127; SD = 0.016; median = 0.130; strong downregulation) (*p* < 0.05), with subsequent decline. While the temporal profiles were comparable, LP treatment consistently induced higher expression values, particularly at 18h (*p* < 0.05).

The *spz4* gene followed a similar trajectory in both treatments, with a pronounced peak at 6h followed by a rapid decline (**Figure 1E**). In LP-treated larvae expression peaked at 6h (M = 1.455; SD = 0.449; median = 1.315), corresponding to mild upregulation (1 < FC ≤ 2), followed by a significant decrease at 12h (M = 0.370; SD = 0.146; median = 0.325; strong downregulation, FC < 0.5) (p < 0.001), and remained low thereafter. Similarly, LK-treated larvae exhibited a peak at 6h (M = 0.990; SD = 0.320; median = 0.925; mild downregulation, 0.5 ≤ FC < 1) and a significant reduction at 12h (M = 0.225; SD = 0.031; median = 0.215; strong downregulation) (*p* < 0.001). Although no statistically significant differences were observed between treatments at corresponding time points, LP consistently elicited higher expression levels, indicating a quantitatively stronger transcriptional response.

The *gallerimycin* expression peaked at 12h in both treatment groups, with consistently higher levels in LP-treated larvae and a progressive decline thereafter (**Figure 1F**). In LP, expression reached its maximum at 12h (M = 39.515; SD = 13.024; median = 34.540), corresponding to strong upregulation (FC > 2), followed by a significant decrease at 18h (M = 30.345; SD = 7.936; median = 31.365; strong upregulation) and 24h (M = 13.087; SD = 2.356; median = 13.140; strong upregulation) (*p* < 0.05). In LK, a peak was also detected at 12h (M = 29.622; SD = 5.044; median = 30.495; strong upregulation), followed by a reduction at later time points, although this decrease was not statistically significant. While the temporal expression profiles were similar between treatments, absolute expression levels were consistently higher in the LP group, with no statistically significant differences between LP and LK at the same time points.

The *gloverin* gene showed early activation in both treatments, with a peak at 6h followed by a rapid decrease (**Figure 1G**). At 6h, expression in the LP group reached M = 19.220 (SD = 3.765; median = 18.950), corresponding to strong upregulation (FC > 2), while LK-treated larvae displayed lower levels (M = 12.885; SD = 3.401; median = 11.590; strong upregulation). No statistically significant differences were detected between treatments at specific time points.

*NADPH oxidase 4-like* expression exhibited distinct temporal profiles between treatments (**Figure 1H**). In LP-treated larvae a significant increase was observed from 3h (M = 1.148; SD = 0.329; median = 1.160; mild upregulation, 1 < FC ≤ 2) to 6h (M = 2.522; SD = 0.295; median = 2.480; strong upregulation, FC > 2) (*p* < 0.05), followed by a further rise to a maximum at 18h (M = 3.700; SD = 1.156; median = 3.605; strong upregulation). Expression levels then slightly declined but remained elevated at 24h. In contrast, in LK-treated larvae, gene expression peaked earlier at 6h (M = 3.962; SD = 1.102; median = 3.555; strong upregulation), following a significant increase compared to 3h (M = 2.350; SD = 0.626; median = 2.310; strong upregulation) (*p* < 0.05). After a slight decrease at 12h (M = 3.365; SD = 1.082; median = 3.340; strong upregulation), a significant drop occurred between 12h and 18h (M = 1.848; SD = 0.618; median = 1.555; mild upregulation) (*p* < 0.05), followed by a secondary increase at 24h. A statistically significant difference between treatments was detected at 18h (*p* < 0.01), with higher expression in the LP group, indicating a more sustained activation over time compared to LK.

The *Hem* gene exhibited similar overall trends in both treatments, although with differences in timing (**Figure 1I**). In the LK group, early activation was evident at 3h (M = 2.605; SD = 0.419; median = 2.740), indicating strong upregulation (FC > 2), followed by a gradual decline. In LP-treated larvae, expression peaked at 6h (M = 2.427; SD = 0.378; median = 2.380; strong upregulation) and then declined significantly at 12h (M = 0.570; SD = 0.188; median = 0.500; mild downregulation, 0.5 ≤ FC < 1) (p < 0.01), with minor fluctuations at later time points. Despite similar temporal profiles, no statistically significant differences were observed between treatments.

Finally, IMPI expression showed substantial differences between treatments (**Figure 1J**). In LP-treated larvae, relatively high early expression was recorded at 3h (M = 0.883; SD = 0.242; median = 0.835), corresponding to mild downregulation (0.5 ≤ FC < 1), followed by a slight decrease at 6 h. A secondary increase occurred at 12h (M = 0.773; SD = 0.220; median = 0.765; mild downregulation), but a marked and statistically significant drop was detected between 12h and 18h (M = 0.065; SD = 0.015; median = 0.065; strong downregulation, FC < 0.5) (*p* < 0.01), which persisted at 24h. Conversely, in LK-treated larvae, expression peaked at 6h (M = 1.722; SD = 0.506; median = 1.645), indicating mild upregulation (1 < FC ≤ 2), followed by a drastic reduction at 12h (M = 0.257; SD = 0.041; median = 0.250; strong downregulation) (p < 0.01). Expression then increased again at 18h (M = 1.013; SD = 0.305; median = 1.040; mild upregulation), before declining significantly at 24h (M = 0.092; SD = 0.028; median = 0.090; strong downregulation) (*p* < 0.01). A significant difference between treatments was observed at 18h (*p* < 0.01), proposing a differential regulation of *IMPI* expression by the two probiotic strains. Overall, LK treatment appeared to elicit a more prolonged and variable expression response over time, whereas LP treatment induced a more transient activation profile.

#### 3.3.2 Spearman’s correlation analysis

##### 3.3.2.1 Gene expression across the two treatments

Time correlation analysis performed on the expression levels of the same genes across the two treatments highlighted both similarities and differences in the temporal modulation of gene expression. The results, summarised in **Table 3**, show that the genes *Dorsal*, *Rel*, *cad*, *spz4*, *gloverin*, and *Hem* display positive and statistically significant correlation coefficients, with ρ (rho) values ranging from 0.812 to 0.969 (*p* < 0.001), indicating a similar temporal expression pattern between LP and LK. The gene *18w* exhibits a moderate correlation (ρ = 0.692, *p* < 0.001). At the same time, *gallerimycin* shows a weaker correlation (ρ = 0.463, *p* < 0.001), leading to a higher degree of variability in the AMPs response. Conversely, no statistically significant correlation was observed for *NADPH oxidase 4-like* (ρ = 0.201, *p = 0.201) and IMPI (ρ = 0.088, p = 0.088*), indicating divergent expression patterns between the two probiotic treatments.

**Table 3:** Spearman correlation coefficients (ρ) assessing the similarity in temporal gene expression patterns of immune-related genes between LP and LK treatments.

Gene	ρ (rho)	P-value	Correlation
*Dorsal*	0.9693	<0.001	Strong
*Rel*	0.8888	<0.001	Strong
*cad*	0.9240	<0.001	Strong
*18w*	0.6917	<0.001	Moderate
*spz4*	0.8115	<0.001	Strong
*gallerimycin*	0.4633	<0.001	Weak
*gloverin*	0.8976	<0.001	Strong
*NADPH oxidase 4*	0.2010	Ns	None
*Hem*	0.8798	<0.001	Strong
*IMPI*	0.0880	Ns	None

Correlations were considered statistically significant when p < 0.05; values with p ≥ 0.05 are reported as ns (not significant). The strength of the correlation was interpreted as follows: none (ρ < 0.3), weak (0.3 ≤ ρ < 0.5), moderate (0.5 ≤ ρ < 0.7), and strong (ρ ≥ 0.7)

##### 3.3.2.2 Gene expression within-treatment

**Figure 2:**
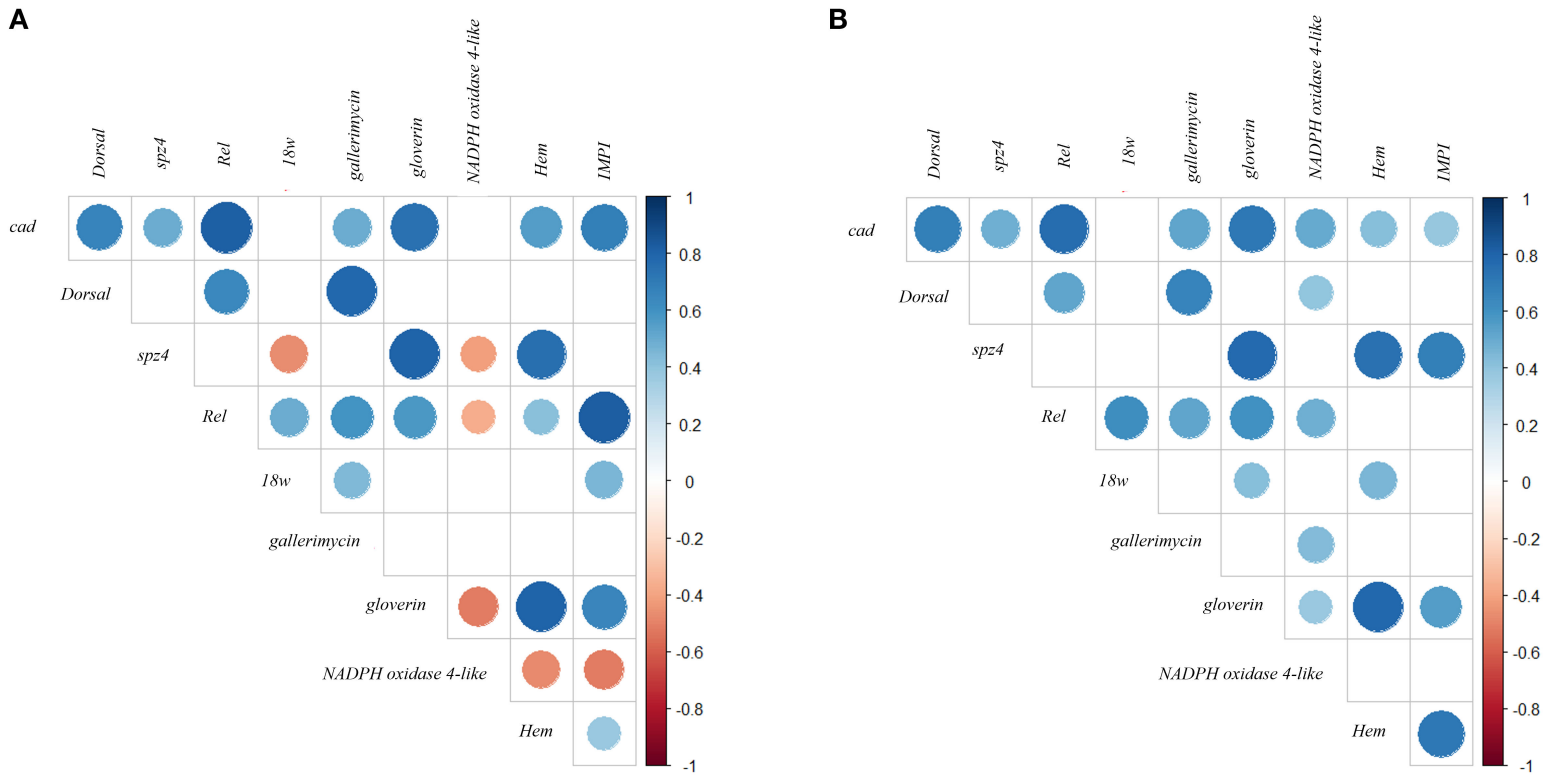
Spearman correlation analysis of temporal gene expression profiles following probiotic treatments. Panels **(A, B)** display the correlations among immune-related genes in *G*. *mellonella* larvae treated with LP and LK, respectively. Spearman correlation coefficients (ρ) were calculated based on gene expression levels measured at five time points (3–24 hours post-treatment). Only statistically significant correlations (p < 0.05) are shown. The size and color intensity of the circles indicate the strength and direction of the correlation (blue = positive, red = negative).

Spearman correlation analysis of temporal gene expression profiles (3–24 h, 5 time points) revealed distinct co-expression patterns among immune-related genes in LP- and LK-treated larvae (**Figure 2**).

###### 3.3.2.2.1 *L. plantarum* ATCC 14917

*G. mellonella* gene expression time correlation matrix following LP treatment, comprising both positive and negative associations, suggests heterogeneous transcriptional responses (**Figure 2A**).

*Dorsal* exhibited a strong positive correlation with *gallerimycin* (ρ = 0.780, *p* < 0.001), and moderate correlations with *Rel* (ρ = 0.641, *p* < 0.001) and *cad* (ρ = 0.666, *p* < 0.001), pointing to a temporal expression profile aligned with both upstream immune regulators and downstream effectors. In contrast, weak and non-significant correlations with *spz4*, *18w*, *gloverin*, *NADPH oxidase 4-like*, *Hem*, and IMPI (ρ < 0.27; p ≥ 0.05) may reflect indirect or asynchronous regulatory interactions.

*Rel* emerged as a central node within the temporal co-expression network of immune-related genes, displaying a broad range of statistically significant correlations across the 3–24h interval. Among the positive associations, strong correlations were observed with *IMPI* (ρ = 0.828, *p* < 0.001) and *cad* (ρ = 0.817, *p* < 0.001). In contrast, moderate correlations were found with *Dorsal* (ρ = 0.640, *p* < 0.001), *gallerimycin* (ρ = 0.599, *p* < 0.001), and *gloverin* (ρ = 0.577, *p* < 0.001). Weaker yet significant correlations were detected with *18w* (ρ = 0.492, *p* < 0.01) and *Hem* (ρ = 0.413, *p* < 0.05). A significant negative correlation was also identified with *NADPH oxidase 4-like* (ρ = –0.362, *p* < 0.05). The only non-significant correlation was observed with *spz4* (ρ = 0.171).

*cad* exhibited a distinct profile of positive temporal correlations with multiple immune-related genes. Statistically significant strong correlations were observed with *Rel* (ρ = 0.817, *p* < 0.001) and *gloverin* (ρ = 0.742, *p* < 0.001). Moderate but significant correlations were identified with *IMPI* (ρ = 0.690, *p* < 0.001), *Dorsal* (ρ = 0.666, *p* < 0.001), and *Hem* (ρ = 0.554, *p* < 0.01), showing a coordinated activation pattern within the analysed temporal window. Additional correlations with *cad* were statistically significant yet of lower magnitude, including *gallerimycin* (ρ = 0.488, *p* < 0.01) and *spz4* (ρ = 0.496, *p* < 0.01). Correlations with *18w* and *NADPH oxidase 4-like* did not reach statistical significance (p ≥ 0.05) and are therefore not shown in **Figure 2A**.

18*wh* showed moderate and statistically significant positive correlations with *Rel* (ρ = 0.492, *p* < 0.001), *gallerimycin* (ρ = 0.446, *p* < 0.05), and *IMPI* (ρ = 0.459, *p* < 0.05). A significant negative correlation was also observed with *spz4* (ρ = –0.462, *p* < 0.05).

*spz4* exhibited a distinct pattern of temporal correlations. Among the statistically significant associations, strong positive correlations were observed with *gloverin* (ρ = 0.800, *p* < 0.001) and *Hem* (ρ = 0.756, *p* < 0.001). In contrast, negative correlations were found with *18w* (ρ = –0.462, *p* < 0.05) and *NADPH oxidase 4-like* (ρ = –0.414, *p* < 0.05). Other correlations did not reach statistical significance.

Furthermore, correlation analysis revealed positive associations between *gallerimycin* and several immune-related genes within the 3–24h interval. The strongest and statistically significant correlation was observed with *Dorsal* (ρ = 0.780, *p* < 0.001), followed by a moderate correlation with *Rel* (ρ = 0.599, *p* < 0.001), and weaker but significant correlations with *cad* (ρ = 0.495, *p* < 0.01) and *18w* (ρ = 0.446, *p* < 0.05). Other correlations were not statistically significant, including weak associations with *Hem* (ρ = 0.265), *NADPH oxidase 4-like* (ρ = 0.244), *IMPI* (ρ = 0.241), and *gloverin* (ρ = 0.160), as well as a non-significant negative correlation with *spz4* (ρ = –0.061).

*Gloverin* displayed one of the most pronounced temporal correlation profiles within the 3–24h interval, with consistent and statistically significant associations. Strong positive correlations were observed with *Hem* (ρ = 0.805, *p* < 0.001), *spz4* (ρ = 0.800, *p* < 0.001), and *cad* (ρ = 0.742, *p* < 0.001), followed by moderate correlations with *IMPI* (ρ = 0.656, *p* < 0.001) and *Rel* (ρ = 0.576, *p* < 0.001). A significant negative correlation was also detected with *NADPH oxidase 4-like* (ρ = –0.515, *p* < 0.01).

*NADPH oxidase 4-like* exhibited a pattern of negative temporal correlations with several immune-related genes over the 3–24h interval. Significant inverse associations were observed with *gloverin* (ρ = –0.515, *p* < 0.01), *IMPI* (ρ = –0.513, *p* < 0.01), *Hem* (ρ = –0.471, *p* < 0.01), *spz4* (ρ = –0.414, *p* < 0.05), and *Rel* (ρ = –0.362, *p* < 0.05).

*Hem* exhibited a well-defined pattern of temporal gene expression correlations during the 3–24h interval, with several statistically significant associations with immune-related genes. Strong positive correlations were observed with *gloverin* (ρ = 0.805, *p* < 0.001) and *spz4* (ρ = 0.756, *p* < 0.001), followed by moderate correlations with *cad* (ρ = 0.553, *p* < 0.01), and weaker correlations with *Rel* (ρ = 0.413, *p* < 0.05) and *IMPI* (ρ = 0.374, *p* < 0.05). A significant negative correlation was also detected with *NADPH oxidase 4-like* (ρ = –0.471, *p* < 0.01). Other non-statistically significant correlations included weak associations with *gallerimycin* (ρ = 0.265, ns), *Dorsal* (ρ = 0.214, ns), and *18w* (ρ = –0.257, ns).

According to the correlation matrix, *IMPI* exhibited statistically significant positive correlations with *Rel* (ρ = 0.828, *p* < 0.001), *cad* (ρ = 0.690, *p* < 0.001), and *gloverin* (ρ = 0.656, *p* < 0.001), along with weaker correlations with *18w* (ρ = 0.459, *p* < 0.05) and *Hem* (ρ = 0.374, *p* < 0.05). A significant negative correlation was also observed with *NADPH oxidase 4-like* (ρ = –0.513, *p* < 0.01), indicating an inverse temporal expression pattern between these genes.

###### 3.3.2.2.2 *L. kefiri* DSM 10551

In LK-treated larvae (**Figure 2B**), *Dorsal* exhibited positive and statistically significant temporal correlations with several immune-related genes. Notably, significant associations were observed with *cad* (ρ = 0.689, *p* < 0.001), *gallerimycin* (ρ = 0.668, *p* < 0.001), *Rel* (ρ = 0.526, *p* < 0.01), and a weaker but still significant correlation with *NADPH oxidase 4-like* (ρ = 0.394, *p* < 0.05). The remaining correlations, while positive, did not reach statistical significance.

These findings highlight *Rel* as a central component in the transcriptional coordination of the LK-induced immune response. Non-significant correlations included weak positive associations with *spz4* (ρ = 0.202, ns) and *Hem* (ρ = 0.263, ns), as well as a weak negative correlation with *IMPI* (ρ = –0.046, ns).

*cad* exhibited a broad and consistent pattern of positive and statistically significant temporal correlations with most immune-related genes within the 3–24h interval. It was significantly correlated with *Rel* (ρ = 0.763, *p* < 0.001), *gloverin* (ρ = 0.718, *p* < 0.001), *Dorsal* (ρ = 0.689, *p* < 0.001), *gallerimycin* (ρ = 0.527, *p* < 0.01), *NADPH oxidase 4-like* (ρ = 0.501, *p* < 0.01), *spz4* (ρ = 0.485, *p* < 0.01), *Hem* (ρ = 0.429, *p* < 0.05), and *IMPI* (ρ = 0.384, *p* < 0.05), indicating a high degree of temporal synchrony in gene expression patterns. The only exception was *18w* (ρ = 0.357, ns), which showed a positive but non-significant correlation, potentially reflecting a more delayed or divergent transcriptional response.

*18w* exhibited selective and statistically significant co-expression with a limited number of immune-related genes. Moderate positive correlations were observed with *Rel* (ρ = 0.610, *p* < 0.001), and weaker but significant associations with *gloverin* (ρ = 0.423, *p* < 0.05) and *Hem* (ρ = 0.456, *p* < 0.05). On the other hand, no statistically significant correlations were found with *cad* (ρ = 0.357, ns), *Dorsal* (ρ = 0.180, ns), *spz4* (ρ = 0.144, ns), *gallerimycin* (ρ = 0.273, ns), *NADPH oxidase 4-like* (ρ = –0.009, ns), and *IMPI* (ρ = –0.001, ns), providing evidence for a more peripheral or asynchronous involvement of *18w* relative to these genes during the transcriptional response.

*spz4* was characterised by a consistent pattern of positive and statistically significant temporal correlations with several immunity-related genes. These included *gloverin* (ρ = 0.777, *p* < 0.001), *Hem* (ρ = 0.743, *p* < 0.001), *IMPI* (ρ = 0.688, *p* < 0.001), and *cad* (ρ = 0.485, *p* < 0.01). For *Rel*, a strong positive temporal correlation was detected with *cad* (ρ = 0.763, *p* < 0.001). Additional moderate associations were observed with *18w* (ρ = 0.610, *p* < 0.001), *gloverin* (ρ = 0.603, *p* < 0.001), *Dorsal* (ρ = 0.526, *p* < 0.01), *gallerimycin* (ρ = 0.529, *p* < 0.01), and *NADPH oxidase 4-like* (ρ = 0.483, *p* < 0.01).

*gallerimycin* exhibited a transcriptional profile characterised by positive temporal correlations with *Dorsal* (ρ = 0.668, *p* < 0.001), *cad* (ρ = 0.527, *p* < 0.01), *Rel* (ρ = 0.529, *p* < 0.01), and *NADPH oxidase 4-like* (ρ = 0.432, *p* < 0.05). Other positive correlations were observed but did not reach statistical significance.

*gloverin* emerged as a central component of a co-expressed immune gene cluster, displaying positive and statistically significant temporal correlations with seven genes: *Hem* (ρ = 0.785, *p* < 0.001), *spz4* (ρ = 0.777, *p* < 0.001), *cad* (ρ = 0.718, *p* < 0.001), *Rel* (ρ = 0.603, *p* < 0.001), *IMPI* (ρ = 0.559, *p* < 0.01), *18w* (ρ = 0.423, *p* < 0.05), and *NADPH oxidase 4-like* (ρ = 0.378, *p* < 0.05). This broad network of associations places *gloverin* at the interface between regulatory elements and effector genes, supporting its key role in the antimicrobial response. In contrast, correlations with *Dorsal* (ρ = 0.184, ns) and *gallerimycin* (ρ = 0.232, ns) were not statistically significant, despite showing a positive trend.

*NADPH oxidase 4-like* exhibited a moderately coordinated expression pattern, with a positive and statistically significant temporal correlation with several immune-related genes. Notable associations included *cad* (ρ = 0.501, *p* < 0.01), *Rel* (ρ = 0.483, *p* < 0.01), *gallerimycin* (ρ = 0.432, *p* < 0.05), *Dorsal* (ρ = 0.394, *p* < 0.05), and *gloverin* (ρ = 0.378, *p* < 0.05).

*Hem* exhibited strong positive temporal correlations with *spz4* (ρ = 0.743, *p* < 0.001), *gloverin* (ρ = 0.785, *p* < 0.001), and *IMPI* (ρ = 0.716, *p* < 0.001). Moderate correlations were also recorded for *cad* (ρ = 0.429, *p* < 0.05) and *18w* (ρ = 0.456, *p* < 0.05).

Finally, *IMPI* displays positive and statistically significant temporal correlations with four immune-related genes: *Hem* (ρ = 0.716, *p* < 0.001), *spz4* (ρ = 0.688, *p* < 0.001), *gloverin* (ρ = 0.559, *p* < 0.01), and *cad* (ρ = 0.384, *p* < 0.05). All non-reported associations were statistically non-significant (*p* > 0.05).

##### 3.3.2.3 Hierarchical clustering analysis across *Lactobacillus* administration

**Figure 3:**
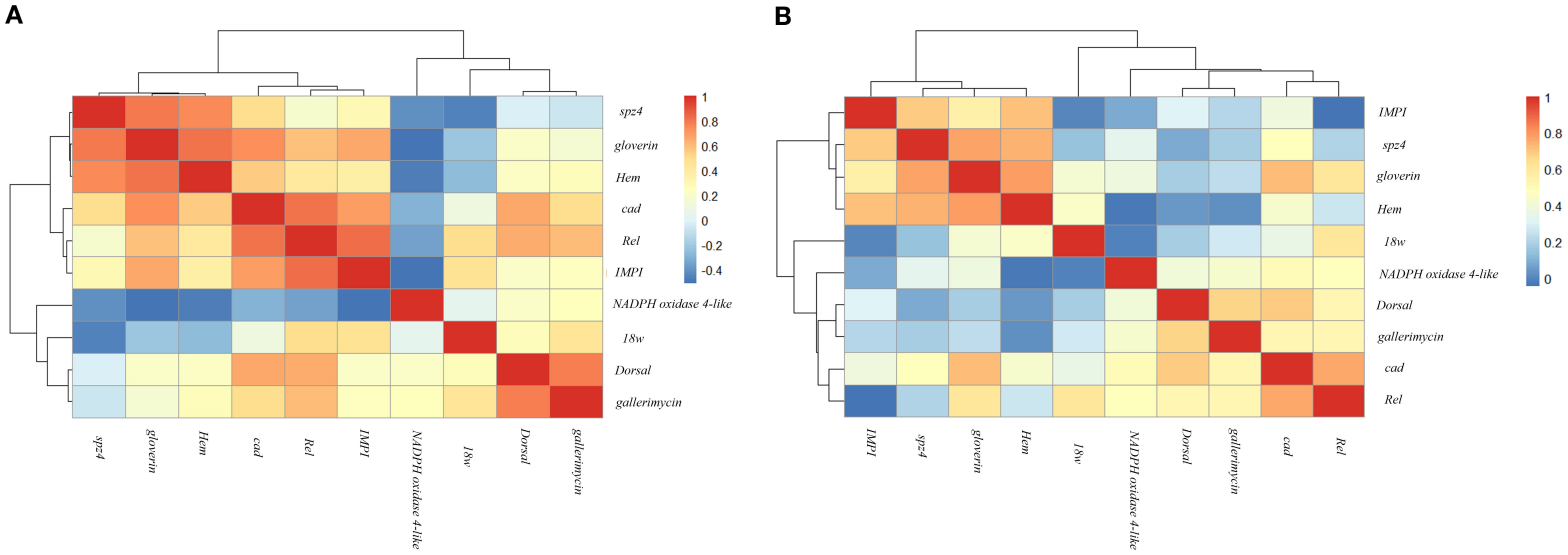
Hierarchical clustering heatmap of Spearman correlation coefficients among immune-related genes following probiotic treatments. Panels **(A, B)** show gene–gene correlations in *G mellonella* larvae treated with LP and LK, respectively, across five time points (3–24 hours post-treatment). The heatmap illustrates the strength and direction of the correlations (ρ) using a color gradient ranging from red (strong positive correlation) to blue (strong negative correlation).

The hierarchical clustering analysis of immune-related gene expression following LP and LK treatments revealed temporally coordinated modules of co-expressed genes (**Figure 3**). The resulting heatmaps enabled the identification of distinct transcriptional patterns, which reflect functional grouping and dynamic regulation across the experimental time points (3–24 hours).

In the LP-treated group (**Figure 3A**), two major gene co-expression clusters were identified, each further subdivided into functionally and temporally coherent subgroups. The first cluster included a subgroup composed of *spz4*, *gloverin*, and *Hem*, which were tightly associated and displayed high correlation coefficients (*spz4*–*gloverin*, ρ = 0.800, *p* < 0.001; *gloverin* –*Hem*, ρ = 0.805, *p* < 0.001; *spz4*–*Hem*, ρ = 0.756, *p* < 0.001). A second subgroup within the same cluster comprised *cad*, *Rel*, and *IMPI*, which exhibited strong mutual correlations (*cad*–*Rel*, ρ = 0.817, *p* < 0.001; *Rel*–*IMPI*, ρ = 0.828, *p* < 0.001; *cad*–*IMPI*, ρ = 0.690, *p* < 0.001). The second major cluster comprised *18w*, *Dorsal*, and *gallerimycin*, which showed moderate correlations (*Dorsal*–*gallerimycin*, ρ = 0.780, *p* < 0.001; *18w* –*gallerimycin*, ρ = 0.446, *p* < 0.05). Within this cluster, *Dorsal* and *gallerimycin* formed a closely linked subgroup, while *18w* branched separately, connecting more distantly to this core pair. Of particular note was *NADPH oxidase 4-like*, which, although grouped within the second cluster, displayed weak or inverse correlations with many genes from the first cluster (e.g., *NADPH oxidase 4-like*–*gloverin*, ρ = –0.515, *p* < 0.01; *NADPH oxidase 4-like* –*IMPI*, ρ = –0.514, *p* < 0.01), indicating a transcriptional profile distinct from the other genes analysed.

In the LK treatment (**Figure 3B**), two main gene co-expression clusters were identified. The first cluster included *spz4*, *gloverin*, and *Hem* group at the same hierarchical level, forming a transcriptionally cohesive module with high pairwise correlation coefficients (*spz4*–*gloverin*, ρ = 0.777, *p* < 0.001; *spz4*–*Hem*, ρ = 0.743, *p* < 0.001; *gloverin* –*Hem*, ρ = 0.785, *p* < 0.001). *IMPI* was associated with this module through a connecting branch and exhibited positive correlations with each of the three genes (*IMPI* –*Hem*, ρ = 0.716, *p* < 0.001; *IMPI* – *spz4*, ρ = 0.689, *p* < 0.001; *IMPI* –*gloverin*, ρ = 0.559, *p* < 0.01). The second cluster was organised into two main subgroups. The first subgroup consisted of *cad* and *Rel*, which were strongly correlated (*cad* – *Rel*, ρ = 0.763, *p* < 0.001). The second subgroup included Dorsal and *gallerimycin*, showing a moderate correlation (Dorsal– *gallerimycin*, ρ = 0.668, *p* < 0.001). These subgroups were connected via a common branch, forming a compact structure within the cluster. *NADPH oxidase 4-like* was linked to this core module through a separate branch. Finally, *18w* was positioned on an independent branch, displaying weaker correlations with the other cluster members.

## 4 Discussion

This study provides compelling evidence supporting the use of *G. mellonella* as a valuable *in vivo* model for investigating host–probiotic interactions, particularly in the context of innate immune responses. By analysing the temporal expression of key immune-related genes following the administration of LP and LK, we identified distinct and strain-specific immunomodulatory patterns. The two probiotics elicited differential dynamics in the timing and amplitude of gene activation, highlighting the utility of this model for the preclinical screening of the immunomodulatory activity of probiotics.

The embryonic polarity protein dorsal from *G. mellonella* aligns with the human transcription factor p65 isoform 1, a canonical regulator of inflammatory signalling.The alignment parameters indicate a statistically significant relationship, with an intermediate level of sequence identity that supports structural conservation. The relatively low coverage suggests that homology is restricted to specific functional regions rather than spanning the entire sequence. The alignment spans the conserved NF-κB/Rel/Dorsal domain (Baeuerle, 1991), as well as structurally preserved regions such as the RHD-n\_RelA domain and the p53-like transcription factor superfamily (Wolfe et al., 1997), highlighting functional conservation of the DNA-binding, dimerisation, and transcriptional activation domains, which are central to immune regulation (**Supplementary Table S1**).

In *G. mellonella*, as in *D. melanogaster*, *Dorsal* plays a central role in antimicrobial peptides activation (AMPs) downstream of the Toll signalling pathway, which is primarily activated in response to fungal or Gram-positive bacterial infections (Sheehan and Kavanagh, 2017). This activation is initiated by the proteolytic cleavage of the *spz4*, which subsequently binds to Toll-like receptors, including canonical Toll and *18w*, triggering a conserved intracellular cascade (Jiang et al., 2010).

In humans, the transcription factor p65 is a central transcriptional activator of pro-inflammatory cytokines, including TNF-α, IL-1β, and IL-6, playing pivotal roles in the response to infection, immune cell activation, and inflammatory resolution (Tak and Firestein, 2001). It is regulated by phosphorylation, ubiquitination, and acetylation; the latter is modulated by SIRT1, which deacetylates p65 to inhibit its activity and control the exacerbation of inflammation, offering a therapeutic avenue in diseases such as diabetes, atherosclerosis, and cancer (Das et al., 2025). NF-κB p65 is also required for maintaining monocyte responsiveness, as platelets have been shown to serve as essential reservoirs of p65 protein in human blood, preventing monocyte immunoparalysis (Hawwari et al., 2024).

From a translational perspective, the modulation of the human transcription factor p65 by *L. plantarum* and *L. kefiri* strengthens the functional link between the insect and human systems. *L. plantarum* strains (e.g., L15, ELF051) have been shown to suppress NF-κB activation through downregulation of the TLR4/MyD88 axis, reducing pro-inflammatory cytokine expression in murine models of colitis and antibiotic-associated diarrhoea (Liang et al., 2023; Yu et al., 2020). These effects are associated with the restoration of gut microbiota composition and suppression of IL-1β and TNF-α. Moreover, *L. plantarum* LLY-606 ameliorates neuroinflammation in ageing mice by inhibiting NF-κB, indicating a systemic immunomodulatory capacity (Shi et al., 2024). Although studies on *L. kefiri* are less extensive, available evidence corroborates similar regulatory effects on the host immune system, promoting IL-10 expression and reducing NF-κB-mediated inflammation in intestinal and systemic contexts (Carasi et al., 2015).

Consistent with these findings, *Dorsal* gene expression across experimental time points revealed notable differences in both the timing and magnitude of transcriptional activation (**Figure 1A**). *Dorsal* exhibited a transient upregulation that peaked at 12 hours post-treatment in both probiotic groups. Although no statistically significant differences were observed between LP and LK treatments at individual time points, LP consistently induced higher absolute expression levels, pointing out a stronger engagement of Toll-mediated immune responses. This may be attributed to differences in microbial-associated molecular pattern (MAMP) recognition or varying capacities for immune priming between the two strains (Sheehan et al., 2018).

The nuclear factor NF-kappa-B p110 subunit isoform X1 in *G. mellonella* is a central component of the IMD signalling pathway, which is classically activated by Gram-negative bacteria through recognition of diaminopimelic acid (DAP)-type peptidoglycan (Sarvari et al., 2020). Although typically associated with defence against Gram-negative bacteria, according to Hedengren-Olcott the induction of AMPs in *D.melanogaster* by *Micrococcus luteus* requires *Relish* (Hedengren-Olcott et al., 2004) This suggests that this protein integrates immune signals from both Gram-negative and Gram-positive challenges, possibly due to a broader ligand recognition spectrum of upstream receptors or functional convergence with Toll pathway outputs. In addition to regulating AMP transcription—including *gloverin*, it is also involved in modulating autophagy-related genes (ATGs), thereby linking humoral and intracellular immune responses, especially in the context of intracellular infections (Kazek et al., 2020).

The *G. mellonella* nuclear factor NF-κB p110 subunit isoform X1 shows significant homology to the human NF-κB p100 subunit isoform a, with a high Bit score and a strongly significant E-value. These results indicate that, despite only moderate overall identity, the two proteins share an evolutionary relationship and retain conserved features characteristic of non-canonical NF-κB signaling (**Supplementary Table S1**).This is reflected in the conservation of several key NF-κB domains: the Rel Homology Domain, responsible for DNA binding and dimerisation; the RHD-n_NFkB2 domain, which corresponds to the N-terminal region of NF-κB2 involved in precursor processing and DNA interaction (Moorthy et al., 2007); and the IPT_NFkappaB domain, a β-sandwich fold that stabilises dimer formation (X. Li and Stark, 2002). Furthermore, nuclear factor NF-kappa-B p110 subunit isoform X1 harbors a p53-like transcription factor fold, providing evidence for roles beyond canonical immunity, including potential involvement in apoptosis, DNA repair, and cellular stress responses (Stroud et al., 2002).

This structural and functional parallel acquires translational significance in light of recent evidence on NF-κB modulation by *L. plantarum*. In murine models, LLY-606 attenuates hyperuricemia-induced inflammation by inhibiting the TLR4/MyD88/NF-κB axis, reducing pro-inflammatory cytokine release and modulating the gut microbiota (Shi et al., 2023). Similarly, L15 exerts anti-inflammatory effects in colitis by suppressing LPS-induced NF-κB activation and lowering IL-6 and TNF-α levels (Yu et al., 2020). In a Parkinson’s disease mouse model, DP189 downregulates NLRP3 and NF-κB signaling, thereby reducing neuroinflammation and influencing the gut–brain axis (L. Wang et al., 2022). Additionally, *L. plantarum* protects against enterotoxigenic *E. coli*-induced intestinal inflammation by inhibiting NF-κB-mediated cytokine cascades, enhancing epithelial barrier integrity (X. Han et al., 2021).

*Rel* gene expression profile revealed a clear peak of transcriptional activation at 12 hours post-probiotic administration, with LP inducing a markedly higher response compared to LK (**Figure 1B**). This pronounced upregulation raising the possibility that LP may activate both the Toll and IMD signalling pathways concurrently, resulting in a coordinated, dual-axis innate immune response in *G. mellonella*—a phenomenon also described in *Drosophila* (De Gregorio et al., 2002) (**Figure 1B**).

The homeobox protein CDX-2 isoform X2 in *G. mellonella* exhibits a high degree of conservation with its human ortholog, homeobox protein CDX-2 isoform 1, as indicated by a Bit score of 137. The remarkably high sequence identity in the aligned regions, despite partial coverage, indicates the presence of highly conserved domains. This findings confirm the functional preservation of the homeodomain, which mediates site-specific DNA interaction in gene promoters involved in epithelial identity and immune regulation. The N-terminal acidic region, also conserved, likely contributes to coactivator recruitment and transcriptional activation (Mannervik, 1999) (**Supplementary Table S1**). *Cad* has been proposed as a negative regulator of the IMD pathway, as its silencing leads to increased expression of antimicrobial peptides (AMPs), implying a role in maintaining immune balance and preventing chronic inflammatory activation (Sarvari et al., 2020).

In humans, CDX2 is a key transcription factor involved in epithelial differentiation, tissue repair, and immune modulation. Its regulation is tightly linked to anti-inflammatory mechanisms, and its dysregulation has been associated with inflammatory and metaplastic disorders (Lu et al., 2020; Sun et al., 2017). Several studies indicate that *L. plantarum* can modulate pathways converging on epithelial differentiation and tissue repair. *L. plantarum* 124 enhances the abundance of beneficial taxa such as *Bifidobacterium* and *Dubosiella*, both associated with homeostatic regulation of epithelial responses (L. Li et al., 2023). Moreover, PS128 promotes serotonin signalling and mucin production (C.-M. Chen et al., 2021).

Following probiotic administration, *cad* exhibited a similar expression profile in both LP- and LK-treated groups, with an early peak at 6 hours, prolonged to 12 hours. However, expression levels were consistently higher in LP-treated larvae, suggesting a more robust early activation of immune signalling. (**Figure 1C**).

The toll-like receptor 7 protein in *G. mellonella* is a Toll-related transmembrane protein characterised by multiple leucine-rich repeat (LRR) domains, a hallmark of molecules involved in innate immune surveillance. These domains form a curved solenoidal structure optimised for protein–protein interactions, which are crucial for ligand recognition, signalling specificity, and receptor stability. The central LRR core is shared across many pattern recognition receptors (PRRs), including Toll-like and SLIT family proteins, underscoring a conserved immune recognition architecture (Ng and Xavier, 2011) (**Supplementary Table S1**).

In *D. melanogaster*, Toll-like receptor 7 is essential for the proper development of the fat body, and its absence in mutant larvae results in impaired induction of a broad range of antimicrobial peptide genes, not limited to those targeting Gram-negative bacteria (Ligoxygakis et al., 2002; Williams et al., 1997), leading to a functional conservation in *G. mellonella*.

The combination of high coverage with low sequence identity suggests a more distant structural similarity, possibly restricted to conserved leucine-rich repeat (LRR) motifs rather than overall functional equivalence (**Supplementary Table S1**). Interestingly, the protein BLAST analysis nevertheless identified the human SLIT1 protein—functionally implicated in immune-related processes—as the top-ranked hit. Slit homolog 1 isa mammalian protein that belongs to the Slit family involved in several key functions in both physiological and pathological processes through interactions with Robo receptors. Initially characterized in the nervous system for their role in axon guidance, Slit/Robo signaling has since been implicated in a broader range of biological activities, including angiogenesis, regulation of immune cell migration, and promotion of tumor cell invasion and metastasis (Tong et al., 2019). Analysis of conserved domains revealed the presence of the LRR domain superfamily (**Supplementary Table S1**). This superfamily is characterized by the presence of LRR domains forming a right-handed β-α superhelix structure, similar to that observed in the bacterial invasion protein internalin (Wollert et al., 2007).

In our time-course analysis, *18w* exhibited a biphasic transcriptional profile in both groups, with initial induction at 3 h, a decline at 6 h, and a secondary rise at 12 h, although expression levels remained below baseline throughout the observation period, indicating downregulation. Larvae treated with LP displayed relatively higher values, particularly at 18 h, compared to LK, being consistent with a more pronounced, albeit sub-threshold, receptor-mediated response (**Figure 1D**).

Protein spaetzle 4, gallerimycin, and gloverin represent key, specialised components of the innate immune system in *G. mellonella*, each fulfilling distinct and highly integrated immune functions.

In insects, spaetzle proteins act as a cytokine-like ligand that, upon proteolytic activation, initiates Toll pathway signalling by binding specific receptors. Although structurally unrelated to vertebrate cytokines (**Supplementary Table S1**), it serves a functionally analogous role by acting as a danger signal that triggers AMP production via Toll-mediated transcriptional cascades (Arnot et al., 2010). Both probiotic groups exhibited a sharp peak in *spz4* expression at 6 h, followed by a rapid decline, with LP consistently showing higher values. Although these levels subsequently dropped below baseline, the overall trend suggests a quantitatively stronger but transient transcriptional activation under LP treatment, despite the predominance of hypoexpression at later time points (**Figure 1E**).

Downstream of this pathway, gallerimycin is a cysteine-rich defensin-like AMP with potent antifungal activity and specificity against Gram-positive bacteria (Schuhmann et al., 2003). Notably, it contains a lipobox-like motif (**Supplementary Table S1**), typically involved in bacterial lipoprotein lipidation and membrane anchoring (Sutcliffe and Harrington, 2002), which may enhance its immune localisation or stability. Though direct lipidation, in insects remains speculative, the motif may confer protease resistance or facilitate interactions with other AMPs (Kovacs-Simon et al., 2011; Nakayama et al., 2012; Zückert, 2014). *Gallerimycin* transcription peaked at 12 h in both groups, with significantly stronger induction in LP-treated larvae, indicating an efficient conversion of upstream signalling into effector output (**Figure 1F**).

In *G. mellonella*, gloverin displays a broader response profile, being induced not only by Gram-negative but also by Gram-positive bacteria and fungi, suggesting a flexible, context-dependent role in immune defence (Serrano et al., 2023). Although it lacks homologous domains with human (**Supplementary Table S1**), gloverin exhibits strong functional similarity to vertebrate cathelicidins such as LL-37, particularly in its capacity to destabilise microbial membranes and influence immune responses (G. Wang, 2014). Its early and robust induction, especially in the LP group, underscores its role as a major effector in systemic immunity (Vogel et al., 2011) (**Figure 1G**).

. Despite a moderate percentage identity, the NADPH oxidase 4-like isoform X1 protein in *G. mellonella* exhibits an extensive coverage and very low E-value with its human counterpart, NADPH oxidase 4 isoform, suggesting strong conservation of core functional domains, particularly the NADPH oxidase catalytic regions (**Supplementary Table S1**). This supports the functional relevance of the *G. mellonella* ortholog in oxidative immune responses.In human NADPH oxidase 4 is a key enzyme in ROS production and redox-mediated innate immunity. Its domain architecture includes a riboflavin synthase-like β-barrel fold, which contributes to stabilising redox-active sites essential for catalytic cycling (**Supplementary Table S1**). These features support the notion that *G. mellonella’s* NOX4-like isoform retains the mechanistic complexity required for ROS-mediated microbial defense, consistent with its proposed functional role (Dym and Eisenberg, 2001).

In *G. mellonella*, as in other insects, NADPH oxidases play a central role in hemocyte-mediated microbial killing during phagocytosis, where the oxidative burst is crucial. Specifically, hemocytes have been shown to produce superoxide during phagocytosis, and its inhibition by diphenyleneiodonium chloride abolishes microbial killing—demonstrating functional NOX activity (Bergin et al., 2005).

In mammals, NADPH oxidase 4 is constitutively expressed in immune and epithelial cells, contributing to host defence as well as redox-sensitive signalling pathways, such as NF-κB, MAPK, and JNK—processes implicated in inflammation, fibrosis, and metabolic disease. Its primary product, H_2_O_2_, serves as a diffusible second messenger in these pathways (Gan et al., 2021).

Although direct interactions with NADPH oxidase 4 were not assessed, *L. plantarum* has been shown to modulate the redox balance in mammalian models. For instance, HFY09 enhances hepatic antioxidant enzymes. It reduces inflammatory cytokines in alcoholic liver disease models (Gan et al., 2021), and restores redox homeostasis in NASH (D. Y. Kim et al., 2023), by an indirect modulation of NOX-dependent pathways. Additional data indicate that *L. plantarum* improves mitochondrial integrity and reduces lipid peroxidation, both of which are linked to NADPH oxidase regulation. Evidence for redox modulation by *L. kefiri* is less extensive. However, CIDCA 8348 reduced TNFα, IL-6, and IL-13 secretion while boosting IL-10 and regulatory T cells in IBD patient-derived cells—changes consistent with redox-sensitive immunoregulation (Curciarello et al., 2021).

In our model, the *expression of NADPH oxidase 4-like* showed a strain-dependent pattern: LP induced a progressive increase, peaking at 18 h, whereas LK resulted in an earlier but less sustained peak at 6 h. The sustained induction by LP suggests prolonged activation of ROS-dependent defences (**Figure 1H**).

The membrane-associated protein Hem in *G. mellonella* exhibits a very strong evolutionary conservationwith human Nck-associated protein 1 isoform 1, with an alignment that covers nearly the entire sequence of both proteins, supporting the hypothesis of functional conservation. Although no Pfam or SMART subdomains are annotated, InterPro analysis links *Hem* to the WAVE regulatory complex, which mediates Rac1-dependent actin polymerisation and cytoskeletal remodelling during phagocytosis (B. Chen et al., 2014) (**Supplementary Table S1**).

In insects, the membrane-associated protein Hem is predominantly expressed in hemocytes and is significantly upregulated during *C. albicans* infection and in response to antifungal treatments such as fluconazole and amphotericin B (Melo et al., 2013). Transcriptomic analyses of immune-challenged larvae further confirm its involvement in cytoskeleton-associated processes, including phagocytosis and stress signalling (Vogel et al., 2011). These data support a model in which *Hem* contributes to actin-driven immune functions, particularly in hemocyte activation and microbial clearance.

In humans, Nck-associated protein 1 plays a crucial role in immune signal transduction and actin cytoskeleton regulation through its interaction with the WAVE complex, facilitating the formation of membrane protrusions essential for phagocytic engulfment (K. A. Han and Ko, 2023). While direct effects of probiotics on Nck-associated protein 1 have not been documented, both *L. plantarum* and *L. kefiri* have demonstrated the ability to enhance phagocytic responses. *L. plantarum* boosts oxidative burst activity and cytokine production in macrophages (Q. Li et al., 2025), and inhibits pathogen adhesion (Saboktakin-Rizi et al., 2021). Similarly, *L. kefiri* improves macrophage function by promoting polarisation and secretion of antimicrobial peptides (Hong et al., 2009).

Following probiotic administration, *Hem* expression peaked earlier in the LK group (3 h), whereas LP induced a delayed but comparable peak at 6 h. These distinct kinetic patterns may reflect strain-specific differences in hemocyte mobilisation or activation (**Figure 1I**).

The inducible metalloproteinase inhibitor protein from *G. mellonella* shows limited sequence similarity to human mucin-6 isoform X1 The low coverage and identity indicate limited homology restricted to short cysteine-rich motifs. While statistically significant, the biological interpretation requires caution, as the relationship likely reflects convergent conservation of disulfide-bonded domains rather than broader functional similarity. (**Supplementary Table S1**). Domain-based analysis reveals the presence of structural motifs commonly associated with extracellular matrix (ECM) protection and protease inhibition. Specifically, *IMPI* contains a serine protease inhibitor domain that effectively blocks proteolytic enzymes involved in tissue degradation. It also harbours a trypsin inhibitor-like cysteine-rich (TIL) domain, whose extensive network of disulfide bridges ensures structural stability and resistance to both microbial and endogenous proteases (Yang et al., 2017). In addition, a fibronectin type I-like domain mediates ECM binding, point to a role in preserving tissue integrity during infection (Smith et al., 1995) (**Supplementary Table S1**).

Consistent with these features, Vilcinskas & Wedde et al. demonstrated that the inducible metalloproteinase inhibitor protein is strongly induced upon microbial challenge, particularly in response to bacterial and fungal metalloproteases such as thermolysin (Vilcinskas and Wedde, 2002). By neutralising these virulence-associated enzymes, it prevents the degradation of epithelial and ECM components, acting as a critical barrier component and amplifying the effectiveness of the insect’s innate immune system (Wedde et al., 1998).

In humans, mucins such as MUC2 and MUC6 form a protective barrier and modulate local immune responses. Several *L. plantarum* strains have been reported to reinforce mucosal defences and reduce inflammation. For example, strain ZDY2013, when combined with isomaltooligosaccharides, enhances the expression of MUC2, tight junction proteins (ZO-1, claudin), and cytokines such as IL-10 and IL-22 in DSS-induced colitis models (Zhang et al., 2024). Although the inducible metalloproteinase inhibitor protein lacks a direct mammalian counterpart, its role in ECM preservation and modulation of host responses parallels the barrier-protective and immunomodulatory functions of mucins, indicating an evolutionary convergence in the strategies employed by insects and mammals to counteract microbial proteases.

*IMPI* gene expression showed one of the most divergent temporal profiles among the genes analysed, with predominantly hypoexpressed levels. LP induced an early but sub-baseline activation at 3 h, followed by a modest increase at 12 h and a marked downregulation after 18 h, indicating a rapid and transient response. Conversely, LK displayed a biphasic profile with a clear overexpression at 6 h, a decrease at 12 h, and a second peak at 18 h, suggesting a more prolonged and dynamic modulation of tissue protection and ECM stabilisation. The marked difference observed at 18h under LK treatment underscores a strain-specific regulation of *IMPI*, leading to a distinctive role in ECM remodeling and immune modulation. This expression pattern reflects LK’s ability to induce transient overexpression followed by delayed reactivation, while LP is characterised by a rapid but short-lived hypoexpressed response (**Figure 1J**).

The temporal expression profiles of individual immune-related genes observed following administration of the two *Lactobacillus* strains were further supported by the results of the correlation analyses. Spearman correlation across the two treatments (**Table 3**) revealed both conserved and divergent patterns of immune gene expression. A high degree of temporal similarity was observed between treatments for several genes, including *cad*, *Dorsal*, *spz4*, *Rel*, *gloverin*, and *Hem*. These findings confirm that core components of the Toll and IMD pathways, as well as effector genes like AMPs and hemocyte markers, follow conserved activation dynamics regardless of probiotic strain. In contrast, *18w* and *gallerimycin* showed moderate or weak correlation, indicating greater inter-strain variability in their expression kinetics. Genes such as *NADPH oxidase 4-like* and *IMPI* did not exhibit significant correlation between treatments, with distinct regulatory responses depending on the strain administered. This divergence could reflect different capacities of LP and LK to modulate oxidative stress and ECM stabilisation pathways, respectively. Moreover, the within-treatment temporal correlation matrices provide additional insight into the coordinated immune responses elicited by each strain. In LP-treated larvae, *Relish* acted as a central node, correlating with *cad*, *IMPI*, *gallerimycin*, and *gloverin*, indicating its role in linking upstream signalling with effector responses. *NADPH oxidase 4-like* showed inverse correlations with several immune genes, notably *gloverin*, *IMPI*, and *Hem*, demonstrating an independently regulated redox-related pathway specific to LP treatment (**Figure 2A**). In contrast, LK treatment induced a broader and more synchronised network, with *cad* significantly correlated with most genes. A tightly connected module involving *spz4*, *gloverin*, *Hem*, and *IMPI* indicated coordinated epithelial and humoral responses (**Figure 2B**).

Finally, the hierarchical clustering confirmed treatment-specific transcriptional modules (**Figure 3**). LP responses included two main clusters—an antimicrobial module (*spz4*, *gloverin*, *Hem*) and a regulatory cluster (*cad*, *Rel*, *IMPI*)—plus a less-connected group (*Dorsal*, *18w*, *gallerimycin*). *NADPH oxidase 4-like* remained isolated (**Figure 3A**), possibly indicating an independent regulatory pathway. In LK-treated larvae, *IMPI* joined the antimicrobial cluster, suggesting enhanced coordination between recognition receptors, effector peptides, and extracellular matrix protection. *NADPH oxidase 4-like* was moderately integrated, reflecting a more cohesive regulatory profile. *18w* remained distinct, with minimal co-expression (**Figure 3B**).

Taken together, these findings demonstrate that *G. mellonella* combines humoral defences (e.g., AMPs, melanisation, oxidative burst) with cellular mechanisms (hemocyte activation and phagocytosis), closely mirroring mammalian innate immune strategies despite structural differences (Sheehan et al., 2018). This evolutionary conservation supports the translational potential of the insect model for screening immunomodulatory effects of probiotics.

To the best of our knowledge, this is the first study to evaluate the immunomodulatory effects of *L. plantarum* ATCC 14917 and *L. kefiri* DSM 10551 in *G. mellonella* larvae without pathogen challenge, focusing exclusively on the host response to probiotic administration. While prior studies have mainly assessed the protective effects and potential toxicity of live probiotics and their derivatives (e.g., cell-free supernatants and bacterial extracellular vesicles) during bacterial or fungal infections (Carmo et al., 2025; Guarnieri et al., 2024; Lam et al., 2025; Marinacci et al., 2025; Rossoni et al., 2017; Santarelli et al., 2025), this study shifts the focus to the direct activation of innate immunity by analysing the temporal expression patterns of ten immune-related genes. These include canonical Toll and IMD pathway components, antimicrobial peptides, oxidative stress mediators, and epithelial integrity markers. Importantly, we demonstrate that gene expression profiles are strain-specific and temporally distinct, supporting the use of this model as a preclinical screening tool for immunomodulatory probiotic properties.

Furthermore, the *in silico* alignment and functional annotation analysis revealed a stratified pattern of homology between *G. mellonella* proteins and their human counterparts. The highest conservation was observed for Hem–NCKAP1 and NOX4-like–NOX4, which showed extensive coverage and strong sequence identity, supporting their relevance in cytoskeletal regulation and redox-mediated immunity. Intermediate levels of conservation were found for NF-κB orthologs (Dorsal–p65, Rel–p100) and CDX2, highlighting preservation of key transcriptional domains. In contrast, proteins such as 18-wheeler (TLR7-like) and IMPI exhibited only limited or domain-restricted similarity. Overall, these results point to a heterogeneous degree of conservation and underline the need for further functional and structural studies to validate these candidates and to identify additional targets relevant for translational immunology. Nevertheless, some genes, such as *spz4*, *gloverin*, and *gallerimycin*, still lack detailed biochemical or functional validation in both insect and mammalian systems, and their interpretation should therefore be approached with caution.

Moreover, the *G. mellonella* model is not intended to replace mammalian *in vivo* testing; rather, it should be considered a cost-effective and ethically advantageous preclinical platform for identifying candidate probiotic strains and elucidating their mechanisms of action before vertebrate experimentation. In conclusion, this study introduces a novel use of *G. mellonella* for investigating immune-related host–microbe interactions, revealing strain-dependent immunomodulation and reinforcing the model’s utility as a platform for functional screening with potential human applicability.

## Data Availability

The datasets presented in this study can be found in online repositories. The names of the repository/repositories and accession number(s) can be found below: https://figshare.com/, https://doi.org/10.6084/m9.figshare.29655497.v1.
