# Supplementary material for: In vivo functional screening on innate immunity of lactic acid bacteria in Galleria mellonella preclinical model: comparative analysis of Lactiplantibacillus plantarum and Lentilactobacillus kefiri
Source: Front Cell Infect Microbiol. 2025 Oct 10;15:1681687. doi: 10.3389/fcimb.2025.1681687 (PMC12549655; doi:10.3389/fcimb.2025.1681687)
Supplement: Supplementary file 1 [file Table1.docx]

***Supplementary Table 1.*** ***In silico* alignment of immune-related proteins from *G. mellonella* with putative human orthologs.**

**Protein sequences from *G. mellonella* were compared with the human proteome using BLASTP. For each protein, the table includes: accession numbers, functional description of the putative human ortholog, alignment metrics (Bit score, query coverage, E-value, and percent identity), and the aligned amino acid region between the query (*G. mellonella* NCBI: txid 7137) and subject (*Homo sapiens* NCBI: txid 9606) sequences. Additionally, InterPro analysis was used to annotate the protein families, conserved domains, and their predicted functions, based on the aligned region. In cases where no significant ortholog was identified in *Homo sapiens*, "N.A." (Not Available) is reported in the corresponding columns.**

| **Gene**  ***G. mellonella*** | **Protein**  ***G. mellonella* (Acc.)** | **Protein**  ***H. sapiens***  **(Acc.)** | **Protein**  ***H. sapiens***  **description** | **Bits Score** | **Query Cover**  **(%)** | **E-value** | **Identity**  **(%)** | **Aligned region**  **(aa)** | **Representative**  **families** | **Representative domains**  **(Pfam/InterPro/SMART)** | **Domains description/**  **functions** |
| --- | --- | --- | --- | --- | --- | --- | --- | --- | --- | --- | --- |
| *Dorsal* | embryonic polarity protein dorsal  (XP_052755011.1) | transcription factor p65 isoform 1  (NP_068810.3) | Canonical NF-κB transcription factor involved in cytokine gene regulation and immune activation in blood immune cells | 242 | 37% | 7e-70 | 45.14% | *G. mellonella*: 58-341  *H. sapiens*: 13-290 | NUCLEAR FACTOR NF-KAPPA-B PROTEIN | NF-kappa-B/Rel/dorsal domain profile (PF00554 / IPR011539) | Composite domain combining DNA-binding and dimerization functions in NF-κB/Rel transcription factors |
|  |  |  |  |  |  |  |  |  |  | Rel homology DNA-binding domain  (PF00554 / IPR011539) | N-terminal domain responsible for sequence-specific DNA recognition (κB motifs) in immune gene regulation |
|  |  |  |  |  |  |  |  |  |  | p53-like transcription factors  (SSF49417 / IPR008967) | Structural superfamily involved in DNA-binding and transcriptional control of cell cycle, apoptosis, and stress responses |
|  |  |  |  |  |  |  |  |  |  | RHD-n_RelA (PF16611 / IPR039833) | N-terminal Rel homology domain variant in RelA; critical for DNA binding and NF-κB complex activation |
|  |  |  |  |  |  |  |  |  |  | E set domains  (part of PF09377 / IPR008967) | Immunoglobulin-like domains stabilizing interactions between NF-κB subunits |
|  |  |  |  |  |  |  |  |  |  | IPT_NFkappaB (PF09377 / IPR008967) | Ig-like β-sandwich domain mediating subunit interactions and dimer interface formation |
|  |  |  |  |  |  |  |  |  |  | Rel homology dimerisation domain  (PF00554 region / IPR011539) | C-terminal half of RHD; promotes dimer formation, essential for DNA-binding specificity and complex stability |
|  |  |  |  |  |  |  |  |  |  | Immunoglobulin-like fold  (IPR013783 | immunoglobulin-like (Ig-like) fold, which consists of a β-sandwich of seven or more strands in two sheets with a Greek-key topology |
| *Rel* | nuclear factor NF-kappa-B p110 subunit isoform X1  (XP_052749400.1) | nuclear factor NF-kappa-B p100 subunit isoform a  (NP_001070962.1) | Immune transcription factor involved in lymphocyte development and non-canonical NF-κB signaling | 179 | 31% | 4e-45 | 37.96% | *G. mellonella*:  63-360  *H. sapiens*:  38-347 | NUCLEAR FACTOR NF-KAPPA-B PROTEIN | p53-like transcription factors  (SSF49417 / IPR008967) | Regulate cell cycle, DNA repair, apoptosis via sequence-specific DNA binding |
|  |  |  |  |  |  |  |  |  |  | Rel homology DNA-binding domain (PF00554 / IPR011539) | Binds κB motifs; N-terminal DNA-binding subdomain of NF-κB/Rel |
|  |  |  |  |  |  |  |  |  |  | NF-κB/Rel/dorsal profile (PF00554 / IPR011539) | Combines DNA-binding + dimerization domains of NF‑κB/Rel factors |
|  |  |  |  |  |  |  |  |  |  | RHD-n_NFkB2 (PF16611 / IPR039833) | N-terminal region of NF‑κB2/p100; involved in p52 processing and DNA binding |
|  |  |  |  |  |  |  |  |  |  | IPT_NFkappaB (E-set) (PF09377 / IPR008967) | Ig-like domain for subunit stability/interactions in NF‑κB |
|  |  |  |  |  |  |  |  |  |  | Rel homology dimerization domain (PF00554 region / IPR011539) | Mediates NF‑κB dimer formation, critical for transcriptional specificity |
|  |  |  |  |  |  |  |  |  |  | Death-like domain superfamily  IPR011029 | required for death signalling, as well as a variety of non-apoptotic functions |

| *cad* | homeobox protein CDX-2 isoform X2  (XP_026765394.1) | homeobox protein CDX-2 isoform 1  (NP_001256.4) | Master transcription factor controlling genes involved in enterocyte identity, proliferation, and immune modulation | 137 | 36% | 6e-38 | 79.07% | *G. mellonella*: 95-180  *H. sapiens*: 163-248 | HOMEOBOX PROTEIN CDX | Caudal like protein activation region | N-terminal acidic region implicated in coactivator recruitment enhancing transcriptional activity |
| --- | --- | --- | --- | --- | --- | --- | --- | --- | --- | --- | --- |
|  |  |  |  |  |  |  |  |  |  | Homeodomain-like  (Pfam: PF00046; InterPro: IPR009057) | Mediates specific binding to DNA sequences in gene promoters |
| *18w* | toll-like receptor 7  (XP_026748858.2) | Slit homolog 1 (*Drosophila*)  (AAI46762.1) | Secreted ROBO ligand that regulates immune cell migration and vascular inflammation | 177 | 61% | 2e-43 | 26.51% | *G. mellonella*:  315-909  *H. sapiens*: 66-678  *G. mellonella*:  133-880  *H. sapiens*: 81-868  *G. mellonella*:  142-667  *H. sapiens*: 293-877 | LRR_TM_DOMAIN-CONTAINING - LEUCINE- | LRR_dom_sf – Leucine-rich repeat domain superfamily (InterPro: IPR011044) | Superfamily of LRR domains mediating protein–protein interactions; common in receptors involved in immunity and development |
|  |  |  |  |  |  |  |  |  |  | Leu-rich_rpt – Leucine-rich repeat (Pfam: PF00560 / InterPro: IPR001611) | Tandem repeat motif forming curved solenoid structures for ligand or protein binding |
|  |  |  |  |  |  |  |  |  |  | Leu-rich_rpt_typical-subtyp – Typical LRR subtype (SMART: LRR_typ / InterPro: IPR003591) | "Typical" LRR motif subtype with conserved spacing; contributes to structural stability in recognition proteins |
|  |  |  |  |  |  |  |  |  |  | Cysteine-rich flanking region, C-terminal (Pfam: PF08316 / SMART: LRRCT / InterPro: IPR003594) | Stabilizes the C-terminal end of LRR domains; important for correct folding and surface exposure |
|  |  |  |  |  |  |  |  |  |  | Leucine-rich repeat N-terminal domain (Pfam: PF01462 / SMART: LRRNT / InterPro: IPR000372) | Caps the N-terminal end of LRR arrays; enhances structural integrity and shields hydrophobic cores |
|  |  |  |  |  |  |  |  |  |  | SRCR domain  IPR001190 | likely to mediate protein-protein interactions and ligand binding |
|  |  |  |  |  |  |  |  |  |  | EGF-like domain  IPR000742 | found in epidermal growth factor |
| *spz4* | protein spaetzle 4  (XP_031764668.1) | N.A. | N.A. | N.A. | N.A. | N.A. | N.A. | N.A. | SPAETZLE/TOLL LIGAND-LIKE | Cystine-knot_cytokine – Cystine-knot cytokine  (Pfam: PF00889 / InterPro: IPR006207) | Structural motif found in growth factors and cytokines; stabilizes protein folding and supports receptor binding in immune and developmental signaling |
|  |  |  |  |  |  |  |  |  |  | Spaetzle  (Pfam: PF06427 / InterPro: IPR009060) | Insect-specific cytokine-like domain; activates Toll pathway via cleavage and binding to Toll receptors, triggering antimicrobial and developmental responses |
| *gloverin* | gloverin  (XP_026764963.2) | N.A. | N.A. | N.A. | N.A. | N.A. | N.A. | N.A. | GLOVERIN-LIKE_PROTEIN - GLOVERIN-LIKE PROTEIN | N.A. | N.A. |
| *gallerimycin* | gallerimycin  (XP_026765221.2) | N.A. | N.A. | N.A. | N.A. | N.A. | N.A. | N.A. | N.A. | Prokaryotic membrane lipoprotein lipid attachment site profile  (Pfam: PF08139 / InterPro: IPR012640 / PROSITE: PS51257). | Recognizes the conserved lipobox motif (L‑(3–7)x‑[G/A/S]‑C) in bacterial lipoprotein precursors; signal peptide is cleaved and lipidated at the cysteine, anchoring the protein to the membrane. |

| *NADPH oxidase 4-like* | NADPH oxidase 4-like isoform X1  (XP_026757760.2) | NADPH oxidase 4 isoform a  (NP_058627.2) | Hydrogen peroxide–producing enzyme expressed in macrophages and endothelial cells; regulates oxidative signaling and innate immune responses | 281 | 73% | 1e-85 | 33.95% | *G. mellonella*:  20-463  *H. sapiens*:  19-503 | NADPH OXIDASE | Fe3_Rdtase_TM_dom – Ferric reductase transmembrane domain  (Pfam: PF01794 / InterPro: IPR002907) | Transmembrane domain involved in electron transfer for ferric iron (Fe³⁺) reduction; essential in iron homeostasis |
| --- | --- | --- | --- | --- | --- | --- | --- | --- | --- | --- | --- |
|  |  |  |  |  |  |  |  |  |  | FAD-binding domain, ferredoxin reductase-type (Pfam: PF00667 / InterPro: IPR0  03097) | Binds FAD cofactor in redox enzymes like ferredoxin-NADP⁺ reductases; mediates electron transfer in oxidative reactions |
|  |  |  |  |  |  |  |  |  |  | Riboflavin synthase-like beta-barrel domain (InterPro: IPR012349) | Structural fold seen in FAD/FMN-binding proteins; supports flavin catalysis and stability |
|  |  |  |  |  |  |  |  |  |  | FAD-bd_8 – FAD-binding domain 8 (Pfam: PF08022 / InterPro: IPR012999) | Specialized FAD-binding motif found in oxidoreductases; enables cofactor attachment in redox reactions |
|  |  |  |  |  |  |  |  |  |  | FNR_nucleotide-bd – Ferredoxin-NADP⁺ reductase nucleotide-binding domain (Pfam: PF03441 / InterPro: IPR005877) | Binds NADP⁺ in FNR-type enzymes; catalyzes electron transfer from ferredoxin/flavodoxin to NADP⁺ |
|  |  |  |  |  |  |  |  |  |  | Ferric reductase, NAD-binding domain (Pfam: PF08030 / InterPro: IPR012999) | NAD(P)H-binding domain involved in ferric iron reduction; couples electron transfer from NAD(P)H to Fe³⁺ |
| *Hem* | membrane-associated protein Hem  (XP_026755739.2) | nck-associated protein 1 isoform 1  (NP_038464.1) | Hematopoietic-specific cytoskeletal adaptor protein involved in immune cell migration, phagocytosis, and immunological synapse formation through regulation of the xc | 1459 | 99% | 0.0 | 61.85% | *G. mellonella*:  3-1114  *H. sapiens*:  1-1122 | SNCK-ASOCIATED_PROTEIN-1 - NCK-ASSOCIATED PROTEIN 1 | N.A. | N.A. |
| *IMPI* | inducible metalloproteinase inhibitor protein  (XP_052753423.1) | mucin-6 isoform X1  (XP_054187951.1) | Mucus-associated glycoprotein involved in epithelial barrier protection | 43.1 | 22% | 0.015 | 22.22% | *G. mellonella*:  616-800  *H. sapiens*:  674-887 | EXTRACELLULAR MATRIX GLYCOPROTEIN RELATED | Serine protease inhibitors (PF00014 / IPR000194) | Inhibit serine proteases; involved in regulation of inflammation, coagulation, and tissue remodeling |
|  |  |  |  |  |  |  |  |  |  | Trypsin inhibitor-like cysteine-rich domain (TIL) (PF01826 / IPR002919) | Cysteine-stabilized fold that blocks protease active sites; contributes to antimicrobial defense and ECM protection |
|  |  |  |  |  |  |  |  |  |  | Fibronectin type I-like domain (FnI-like)  (PF00041 / IPR000782) | Mediates interaction with extracellular matrix components; supports tissue structure and cell adhesion |
